# Targeting p21-activated kinase 4 (PAK4) with pyrazolo[3,4-*d*]pyrimidine derivative SPA7012 attenuates hepatic ischaemia-reperfusion injury in mice

**DOI:** 10.1080/14756366.2022.2106478

**Published:** 2022-08-03

**Authors:** Yuancheng Mao, Eun Lee, Xiaohui Yang, Eun Ju Bae, Raok Jeon, Byung-Hyun Park

**Affiliations:** a Department of Biochemistry and Research Institute for Endocrine Sciences, Jeonbuk National University Medical School, Jeonju, Republic of Korea; b Research Institute of Pharmaceutical Sciences, College of Pharmacy, Sookmyung Women's University, Seoul, Republic of Korea; c School of Pharmacy, Jeonbuk National University, Jeonju, Republic of Korea

**Keywords:** PAK4, pyrazolo[3,4-*d*]pyrimidine, liver, ischaemia-reperfusion, hypoxia-reoxygenation, Nrf2

## Abstract

p21-Activated kinase 4 (PAK4), one of the serine/threonine kinases activated by Rho-family GTPases, has been widely studied as an oncogenic protein that is overexpressed in many types of cancers. In our recent study, PAK4 upregulation was observed in mice exhibiting hepatic ischaemia-reperfusion (I/R) and in liver transplantation patients. Liver I/R injury was also attenuated in *Pak4* KO mice. Herein, we report a novel series of pyrazolo[3,4-*d*]pyrimidine derivatives of type I ½ PAK4 inhibitors. The most potent compound SPA7012 was evaluated to determine the pharmacological potential of PAK4 inhibitor in I/R injury in mice. Mice with I/R injury showed typical patterns of liver damage, as demonstrated by increases in serum levels of aminotransferases and proinflammatory cytokines, hepatocellular necrosis and apoptosis, and inflammatory cell infiltration, relative to sham mice. Conversely, intraperitoneal administration of SPA7012 dramatically attenuated biochemical and histopathologic changes. Mechanistically, stabilisation of nuclear factor-erythroid 2-related factor 2 (Nrf2), a master regulator of anti-oxidative response, was observed following SPA7012 treatment. SPA7012 treatment in primary hepatocytes also attenuated hypoxia-reoxygenation-induced apoptotic cell death and inflammation. Together, these results provide experimental evidence supporting the use of PAK4 inhibitors for alleviation of I/R-induced liver damage.

## 1. Introduction

Hepatic ischaemia/reperfusion (I/R) injury following surgical procedures such as liver tumour resection and liver transplantation is a major cause of liver dysfunction and even liver failure.^1^ The primary pathogenic mechanism of hepatic I/R injury is cell death and inflammation. The initial hypoxic injury due to the interruption of blood flow causes parenchymal cell death.^2^ During the reperfusion period, the return of blood flow further aggravates liver injury due to an inflammatory response, including macrophage and neutrophil infiltration, and cytokine/chemokine production.^3^ These events together with reoxygenation produce reactive oxygen species (ROS), contributing to further cellular dysfunction and damage.

To protect against ROS-induced tissue damage, liver cells develop defense mechanisms including coordinated induction of various antioxidant proteins. NF-E2-related factor 2 (Nrf2) is a prime example of this response.^4^^,^^5^ Under basal conditions, Nrf2 is sequestered in the cytoplasm by binding to its repressor, Kelch-like ECH-associated protein 1 (Keap1). Oxidative stress modifies cysteine residues of Keap1, resulting in dissociation of Nrf2 from Keap1 and subsequent translocation from the cytoplasm to the nucleus. Nuclear Nrf2 binds to the antioxidant response element (ARE) present in the promoter regions of antioxidant enzymes, such as haem oxygenase-1 (HO-1), NAD(P)H quinone oxidoreductase 1 (NQO1), and glutathione peroxidase (GPx).^6^ In this process, multiple protein kinases participate in the subcellular localisation of Nrf2 and its protein stability. For example, protein kinase C, casein kinase 2, and AMP-activated kinase phosphorylate different regions of Nrf2 and promote nuclear translocation of Nrf2. Conversely, glycogen synthase kinase 3β and Fyn-mediated phosphorylation shift the subcellular distribution of Nrf2 towards the cytoplasm where it is degraded via the ubiquitin-proteasome pathway.^7^^,^^8^ Recently, we have reported that p21-activated kinase 4 (PAK4), an oncogenic protein, phosphorylates Nrf2 at T369 and exports Nrf2 from the nucleus to the cytoplasm.^9^ In contrast, genetic deletion of *Pak4* increases protein stability of Nrf2 and suppresses oxidative stress. These results indicate that PAK4 inhibition is an effective way to enable the Nrf2-mediated antioxidant response to protect mice from I/R injury.

Several PAK4 inhibitors have been described over the last decade. PF-3758309 was first developed by Pfizer as a pan-PAK inhibitor through structure-based design combined with high-throughput screening and displayed anti-tumour effects against several types of cancers.^10^^,^^11^ PF-3758309 was evaluated in clinical trials (NCT00932126); however, further investigation was terminated due to undesirable pharmacokinetic properties and poor bioavailability.^12^ A second compound, KPT-9274, a dual inhibitor of PAK4 and nicotinamide phosphoribosyltransferase,^13^ is currently in clinical trials (NCT02702492) for the treatment of patients with refractory/relapsed haematologic and solid tumours. Recently, Guo et al. developed PAK4 inhibitor as a prodrug form (CZh-226) and observed improved pharmacokinetic and tissue distribution and well tolerability in a rat model of cancers.^13^^,^^14^ Unfortunately, use of the aforementioned PAK4 inhibitors has been limited by their weak potency and lack of PAK4 selectivity. Based on our previous study confirming PAK4 as a negative regulator of Nrf2-ARE pathways,^9^ we closely examined our newly identified PAK4-specific inhibitor SPA7012 for application as a therapeutic for the treatment of hepatic I/R injury. We further examined the molecular basis underlying the protective activities of this small molecule against oxidative stress in cultured hepatocytes with hypoxia-reoxygenation (H/R) and in mice with hepatic I/R.

## 2. Experimental section

### 2.1. Chemistry

#### 2.1.1. General

Most reagents and solvents were purchased from commercial sources and used without further purification. All reactions were performed within a nitrogen or argon atmosphere with anhydrous solvents. To monitor the reaction, thin-layer chromatography was performed using Kieselgel 60 F254 plates (Merk Millipore, MA, USA). Flash column chromatography was performed using silica gel 60 (230–400 mesh, Merck Millipore) with the indicated solvents. Medium pressure liquid chromatography was performed with Combiflash® Rf 200 with RediSep Rf columns (Teledyne ISO, Hunt Valley, MD, USA). NMR spectra were recorded on a Varian YH 400 spectrometer (^1^H 400 MH z, ^13 ^C 100 MHz, Agilent, Palo Alto, CA, USA) and an Avance III HD 500 spectrometer (^1^H 500 MHz, ^13 ^C 125 MHz, Bruker, Billerica, MA, USA). Chemical shifts are expressed as δ units using tetramethylsilane as the external standard.

#### 2.1.2. Synthesis

##### 2.1.2.1. 2–(1-Methoxyethylidene)malononitrile^1^

A mixture of malononitrile (5.0 mL, 78.60 mmol) and trimethyl orthoacetate (11.0 mL, 86.46 mmol) was heated to reflux for 4 h. After cooling to room temperature, the reaction mixture was purified using MPLC with a gradient of 0–30% EtOAc in *n*-hexane to provide compound **2** (9.51 g, 99%) as a light yellow liquid: *R*_f_ = 0.30 (*n*-hexane:EtOAc = 3:2); ^1^H NMR (400 MHz, CDCl_3_) δ 4.08 (s, 3H), 2.41 (s, 3H); ^13 ^C NMR (100 MHz, CDCl_3_) δ 185.7, 113.2, 111.1, 66.3, 58.6, 17.4.

##### 2.1.2.2. 2–(3-Dimethylamino-1-methoxyallylidene)malononitrile^2^

To a mixture of compound **1** (9.51 g, 77.86 mmol) in MeOH (120 mL), DMF-DMA (11.7 mL, 85.65 mmol) was added. The reaction mixture was heated at reflux for 1 h. After cooling to room temperature, the reaction mixture was concentrated and diluted with water. The resulting mixture was extracted with dichloromethane (DCM). The combined organic layers were washed with brine and dried over MgSO_4_, filtered, and concentrated *in vacuo*. The residue was recrystallised (EtOH 100%) and purified by flash column chromatography to provide compound **3** (14.60 g, 67%) as a brown solid: *R*_f_ = 0.19 (*n*-hexane:EtOAc = 1:1); ^1^H NMR (400 MHz, CDCl_3_) δ 7.55 (d, *J* = 12.3 Hz, 1H), 5.09 (d, *J* = 12.3 Hz, 1H), 4.10 (s, 3H), 3.21 (s, 3H), 2.95 (s, 3H); ^13 ^C NMR (100 MHz, CDCl_3_) δ 181.7, 152.8, 117.4, 116.1, 87.4, 60.7, 45.8, 37.4.

##### 2.1.2.3. 2-Bromo-4-methoxynicotinonitrile^3^

To a mixture of compound **2** (2.30 g, 12.96 mmol) in 48% HBr (26 mL), acetic acid glacial (13 mL) was added. After stirring for 14 h at room temperature, the reaction mixture was neutralised with NaHCO_3_ (aq) and extracted with EtOAc. The combined organic layers were washed with brine and dried over MgSO_4_, filtered, and concentrated *in vacuo*. The residue was purified by medium pressure liquid chromatography (MPLC) with a gradient of 0–40% EtOAc in *n*-hexane to provide compound **3** (1.81 g, 66%) as a yellow solid: *R*_f_ = 0.30 (*n*-hexane:EtOAc = 1:1); ^1^H NMR (400 MHz, CDCl_3_) δ 8.40 (d, *J* = 5.9 Hz, 1H), 6.92 (d, *J* = 5.9 Hz, 1H), 4.03 (s, 3H); ^13 ^C NMR (100 MHz, CDCl_3_) δ 168.7, 153.5, 145.3, 113.3, 106.1, 103.8, 57.0.

##### 2.1.2.4. 4-Hydroxy-1*H*-pyridin-2-one^4^

A reaction tube was charged with concentrated hydrochloric acid (HCl) (6 mL) solution and then compound **3** (1.38 g, 6.48 mmol) was added. The tube was sealed, and the mixture was heated at 160 °C for 15 h. After cooling to room temperature, the reaction mixture was diluted with water and neutralised with 2 N NaOH (aq). The resulting mixture was dried over reduced pressure and dissolved in MeOH. The resulting suspension was filtered, and the filtrate was purified by MPLC with a gradient of 0–20% MeOH in CHCl_3_ to provide compound **4** (676 mg, 94%) as a white solid: *R*_f_ = 0.22 (CHCl_3_:MeOH = 8:1); ^1^H NMR (400 MHz, DMSO-d_6_) δ 11.07 (s, 1H), 7.26 (d, *J* = 7.2 Hz, 1H), 5.88 (d, *J* = 7.2 Hz, 1H), 5.60 (s, 1H), 3.12 (s, 1H); ^13 ^C NMR (100 MHz, DMSO-d_6_) δ 168.5, 164.9, 136.2, 100.5, 99.1.

##### 2.1.2.5. 2,5-Dihydropyrido[4,3-*b*]indolone^5^

To a mixture of compound **4** (312 mg, 2.81 mmol) in diphenyl ether (28 mL), phenylhydrazine (0.34 mL, 3.37 mmol) was added. The reaction mixture was heated at reflux in a pre-heated sand bath and stirred for 18 h. After cooling to room temperature, the reaction mixture was poured onto *n*-hexane (300 mL). The precipitate was filtered and rinsed with tetrahydrofuran (THF). The combined filtrate was purified by flash column chromatography to provide **5** (101 mg, 20%) as a brown solid: R*_f_* = 0.33 (CHCl_3_:MeOH = 8:1); ^1^H NMR (400 MHz, CD_3_OD) δ 8.22 (d, *J* = 7.8 Hz, 1H), 7.47 (d, *J* = 8.1 Hz, 1H), 7.36 (d, *J* = 7.1 Hz), 7.34 (t, *J* = 8.1 Hz, 1H), 7.24 (t, *J* = 7.8 Hz, 1H), 6.68 (d, *J* = 7.1 Hz, 1H).

##### 2.1.2.6. 1-Chloro-5*H*-pyrido[4,3-*b*]indole^6^

A mixture of compound **5** (648 mg, 3.52 mmol) and phosphorous oxychloride (10 mL) was heated to reflux for 14 h. After cooling to room temperature, the reaction mixture was concentrated, neutralised with NaHCO_3_ (aq) and extracted with DCM. The combined organic layers were washed with brine and dried over MgSO_4_, filtered, and concentrated *in vacuo*. The residue was purified by flash column chromatography to provide compound **6** (376 mg, 53%) as a pale yellow solid: R*_f_* = 0.51 (CHCl_3_:MeOH = 10:1); ^1^H NMR (500 MHz, DMSO-d_6_) δ 12.15 (s, 1H), 8.38 (d, *J* = 7.9 Hz, 1H), 8.24 (d, *J* = 5.6 Hz, 1H), 7.65 (d, *J* = 7.5 Hz, 1H), 7.57 (t, *J* = 7.5 Hz, 1H), 7.54 (d, *J* = 5.6 Hz, 1H), 7.37 (t, *J* = 7.9 Hz, 1H); ^13 ^C NMR (125 MHz, DMSO-d_6_) δ 145.9, 144.2, 144.1, 140.1, 127.7, 122.4, 121.1, 120.0, 116.7, 112.3, 107.0.

##### 2.1.2.7. 4-Methoxybenzyl(5*H*-pyrido[4,3-*b*]indol-1-yl)amine^7^

A reaction tube was charged with compound **6** (407 mg, 2.01 mmol) and 4-methoxybenzylamine (5.3 mL, 40.12 mmol). The tube was sealed, and the mixture was heated at 200 °C for 4 h. After cooling to room temperature, the reaction mixture was diluted with water and extracted with EtOAc. The combined organic layers were washed with brine and dried over MgSO_4_, filtered, and concentrated *in vacuo*. The residue was purified by flash column chromatography to provide compound **7** (760 mg, quantitative) as a pale yellow solid: R*_f_* = 0.32 (*n*-hexane:EtOAc = 1:1); ^1^H NMR (400 MHz, CDCl_3_) δ 8.76 (s, 1H), 8.12 (dd, *J* = 5.6, 2.0 Hz, 1H), 7.76 (d, *J* = 8.0 Hz, 1H) 7.49–7.33 (m, 3H), 7.30–7.20 (m, 2H), 6.96–6.84 (m, 2H), 6.78 (dd, *J* = 5.6, 2.0 Hz, 1H), 5.13 (s, 1H), 4.87(d, *J* = 3.2 Hz, 2H), 3.80 (s, 3H).

##### 2.1.2.8. (5–(3-Bromophenyl)-5*H*-pyrido[4,3-*b*]indolyl)(4-methoxybenzyl)amine^8^

To a mixture of compound **7** (674 mg, 2.22 mmol), copper powder (283 mg, 4.45 mmol), potassium carbonate (922 mg, 6.67 mmol), 18-crown-6 (178 mg, 0.67 mmol) in 1,2-dichlorobenzene (10 mL), was added 3-bromo-1-iodobenzene (434 μl, 3.33 mmol). The reaction mixture was heated at 180 °C for 5 h. The hot reaction mixture was filtered through a celite pad and rinsed with hot EtOAc. The combined filtrate was washed with brine and dried over MgSO_4_, filtered, and concentrated *in vacuo*. The residue was purified by flash column chromatography to provide compound **8** (436 mg, 43%) as a yellow solid: *R*_f_ = 0.63 (*n*-hexane:EtOAc = 2:1); ^1^H NMR (400 MHz, CDCl_3_) δ 8.12 (s, 1H), 7.81 (s, 1H) 7.72–7.58 (m, 2H), 7.52–7.27 (m, 7H), 6.92 (d, *J* = 6.8 Hz, 2H), 6.70 (s, 1H), 5.19 (s, 1H), 4.87(s, 2H), 3.81 (s, 3H).

##### 2.1.2.9. 5–(3-Bromophenyl)-5*H*-pyrido[4,3-*b*]indolylamine^9^

A mixture of compound **8** (391 mg, 0.85 mmol) and trifluoroacetic acid (3.3 mL) was heated to 60 °C for 30 min. After cooling to room temperature, the reaction mixture was poured onto ice water, neutralised with NaHCO_3_ (aq) and extracted with EtOAc. The combined organic layers were washed with brine and dried over MgSO_4_, filtered, and concentrated *in vacuo*. The residue was purified by flash column chromatography to provide compound **9** (336 mg, quantitative) as a pale yellow solid: *R*_f_ = 0.11 (*n*-hexane:EtOAc = 1:1); ^1^H NMR (400 MHz, CDCl_3_) δ 8.00 (d, *J* = 5.6 Hz, 1H), 7.92 (d, *J* = 7.2 Hz, 1H), 7.67 (s, 1H), 7.63 (d, *J* = 7.2 Hz, 1H), 7.53–7.28 (m, 5H), 6.72 (t, *J* = 5.2 Hz, 1H), 5.47 (s, 2H).

##### 2.1.2.10. General procedures for the synthesis of compounds 10a–c, 14a–d and 18a–b

To a mixture of compound **9**, **13** or **17** (1.0 equiv.) in TEA/DMSO (2:1) was added bis(triphenylphosphine)palladium(II) dichloride (1.0 equiv.) under a nitrogen atmosphere. After stirring at room temperature for 10 min, alkyl acetylene (10.0 equiv.) was added. The reaction mixture was heated at 70 °C for 1 h. After cooling to room temperature, the mixture was filtered through a celite pad and rinsed with EtOAc. Water was added to the filtrate and the mixture was extracted with EtOAc. The combined organic layers were dried over MgSO_4_, filtered, and concentrated *in vacuo*. The residue was purified by flash column chromatography (CHCl_3_:EtOAc or EtOAc:MeOH) to provide compounds **10**, **14** or **18**.

##### 2.1.2.11. 4–(3–(1-Aminopyrido[4,3-*b*]indol-5-yl)phenyl)-2-methylbutyn-2-ol (10a)

Off-white solid (71%): *R*_f_ = 0.31 (EtOAc 100%); ^1^H NMR (400 MHz, CDCl_3_) δ 7.97 (d, *J* = 5.8 Hz, 1H), 7.87 (d, *J* = 7.9 Hz, 1H), 7.54–7.48 (m, 3H), 7.40–7.31 (m, 4H), 6.67 (d, *J* = 5.8 Hz, 1H), 5.35 (s, 2H), 3.56 (s, 1H), 1.65 (s, 6H); ^13 ^C NMR (100 MHz, CDCl_3_) δ 153.9, 146.4, 143.5, 139.8, 136.6, 131.3, 130.0, 129.9, 126.8, 125.2, 125.0, 121.8, 121.4, 120.3, 110.0, 104.2, 97.8, 96.0, 80.6, 65.2, 31.4; HRMS m/z: 342.1614 [M + H]^+^; calculated C_22_H_20_N_3_O^+^: 342.1606.

##### 2.1.2.12. 3-(1-Aminopyrido[4,3-b]indol-5-yl)phenylethynylcyclohexan-1-ol (10 b)

Yellow solid (49%): *R*_f_ = 0.38 (EtOAc 100%); ^1^H NMR (500 MHz, CDCl_3_) δ 7.99 (d, *J* = 5.9 Hz, 1H), 7.90 (d, *J* = 7.6 Hz, 1H), 7.60–7.51 (m, 3H), 7.46–7.33 (m, 4H), 6.71 (d, *J* = 5.9 Hz, 1H), 5.42 (s, 2H), 4.01 (s, 1H), 2.07–1.99 (m, 2H), 1.80–1.53 (m, 7H), 1.35–1.24 (m, 1H); ^13 ^C NMR (125 MHz, CDCl_3_) δ 153.9, 146.5, 143.3, 139.9, 136.7, 131.5, 130.1, 130.0, 127.0, 125.3, 125.2, 121.8, 121.6, 120.4, 110.1, 104.3, 98.0, 94.9, 82.9, 68.9, 40.0, 25.2, 23.4; HRMS m/z: 382.1930 [M + H]^+^; calculated C_25_H_24_N_3_O^+^: 382.1919.

##### 2.1.2.13. 4–(3-(1-Aminopyrido[4,3-*b*]indol-5-yl)phenyl)-2-methylbutyn-1,2-diol (10c)

Off-white solid (10%): *R*_f_ = 0.16 (EtOAc 100%); ^1^H NMR (500 MHz, CD_3_OD) δ 8.24 (d, *J* = 7.5 Hz, 1H), 7.88 (d, *J* = 6.1 Hz, 1H), 7.70–7.63 (m, 3H), 7.55 (dt, *J* = 7.0, 2.1 Hz, 1H), 7.49–7.36 (m, 3H), 6.73 (d, *J* = 6.1 Hz, 1H), 3.61 (s, 2H); ^13 ^C NMR (125 MHz, CD_3_OD) δ 154.1, 146.5, 141.9, 139.8, 136.6, 131.3, 130.1, 129.8, 126.9, 125.2, 125.1, 121.8, 121.5, 120.6, 109.5, 103.8, 97.0, 93.3, 81.7, 69.6, 68.2, 24.7; HRMS m/z: 358.1561 [M + H]^+^; calculated C_22_H_20_N_3_O_2_^+^: 358.1556.

##### 2.1.2.14. 5-Amino-3-methyl-1H-pyrazole-4-carbonitrile^11^

To a solution of compound **1** (1.64 g, 13.42 mmol) in EtOH (25 mL), hydrazine monohydrate (1.3 mL, 26.84 mmol) was added. The reaction mixture was heated at 80 °C for 1 h. After cooling to room temperature, the reaction mixture was concentrated. The residue was purified by flash column chromatography to provide compound **11** (1.60 g, 98%) as an off-white solid: *R*_f_ = 0.31 (*n*-hexane:EtOAc = 1:2); ^1^H NMR (500 MHz, CD_3_OD) δ 2.25 (s, 3H).

##### 2.1.2.15. 3-Methyl-1H-pyrazolo[3,4-*d*]pyrimidin-4-ylamine^12^

A mixture of compound **11** (1.37 g, 11.22 mmol) and formamide (9 mL) was heated to 180 °C for 18 h. After cooling to room temperature, the reaction mixture was poured into water and cooled via ice bath. The solid was filtered and rinsed with water and MeOH to provide compound **12** (1.10 g, 65%) as a yellow solid without further purification: *R*_f_ = 0.26 (EtOAc:MeOH = 10:1); ^1^H NMR (500 MHz, CD_3_OD) δ 8.15 (s, 1H), 2.62 (s, 3H); ^13 ^C NMR (125 MHz, CD_3_OD) δ 164.7, 159.0, 155.5, 152.0, 98.6, 12.9.

##### 2.1.2.16. General procedures for the synthesis of compounds 13 and 17a–b

To a mixture of compound **12** or **16** (1.0 equiv.), copper(I) iodide (0.2 equiv.), potassium carbonate (2.0 equiv.) in DMF, DMEDA (0.4 equiv.) and 3-bromo-1-iodobenzene (1.2 equiv.) were added. The reaction mixture was heated at 110 °C for 3 to 23 h. After cooling to room temperature, the reaction mixture was filtered through a celite pad and rinsed with EtOAc. The combined filtrate was washed with brine and dried over MgSO_4_, filtered, and concentrated *in vacuo*. The residue was purified by flash column chromatography (EtOAc:MeOH) to provide compound **13** or **17**.

##### 2.1.2.17. (3-Bromophenyl)-3-methyl-1H-pyrazolo[3,4-*d*]pyrimidin-4-ylamine^13^

Off-white solid (25%): *R*_f_ = 0.67 (EtOAc:MeOH = 10:1); ^1^H NMR (500 MHz, CD_3_OD) δ 8.40 (s, 1H), 8.28 (s, 1H), 8.18 (d, *J* = 8.0 Hz, 1H), 7.49–7.40 (m, 2H), 2.69 (s, 3H); ^13 ^C NMR (125 MHz, CD_3_OD) δ 158.9, 156.2, 154.4, 143.8, 142.2, 140.0, 130.2, 128.6, 123.5, 122.0, 119.4, 13.1.

##### 2.1.2.18. 4–(3–(4-Amino-3-methylpyrazolo[3,4-*d*]pyrimidin-1-yl)phenyl)-2-methylbutyn-2-ol (14a, SPA7011)

Pale yellow solid (58%): *R*_f_ = 0.29 (CHCl_3_:EtOAc = 1:3); ^1^H NMR (500 MHz, CD_3_OD) δ 8.26 (s, 1H), 8.18 (s, 1H), 8.08 (d, *J* = 8.2 Hz, 1H), 7.48 (t, *J* = 8.0 Hz, 1H), 7.36 (d, *J* = 7.7 Hz, 1H), 2.69 (s, 3H), 1.61 (s, 6H); ^13 ^C NMR (125 MHz, CD_3_OD) δ 158.9, 156.1, 154.1, 143.5, 138.8, 128.8, 123.9, 123.8, 120.9, 100.5, 94.5, 80.5, 64.5, 30.3, 13.1; HRMS m/z: 308.1513 [M + H]^+^; calculated C_17_H_18_N_5_O^+^: 308.1511.

##### 2.1.2.19. 3-(4-Amino-3-methylpyrazolo[3,4-*d*]pyrimidin-1-yl)phenylethynylcyclohexan-1-ol (14 b, SPA7012)

Pale yellow solid (85%): *R*_f_ = 0.33 (CHCl_3_:EtOAc = 1:3); ^1^H NMR (500 MHz, CD_3_OD) δ 8.14 (s, 1H), 8.06 (s, 1H), 7.98 (d, *J* = 8.0 Hz, 1H), 7.37 (dd, *J* = 8.0, 7.7 Hz, 1H), 7.26 (d, *J* = 7.7 Hz, 1H), 2.57 (s, 3H), 1.96–1.84 (m, 2H), 1.71–1.45 (m, 7H), 1.29–1.16 (m, 1H); ^13 ^C NMR (125 MHz, CD_3_OD) δ 158.9, 156.1, 154.2, 143.5, 138.8, 128.9, 128.8, 124.0, 123.8, 120.9, 100.5, 93.6, 68.0, 39.5, 25.0, 23.0, 13.1; HRMS m/z: 348.1827 [M + H]^+^; calculated C_20_H_22_N_5_O^+^: 348.1824

##### 2.1.2.20. 4–(3-(4-Amino-3-methylpyrazolo[3,4-*d*]pyrimidin-1-yl)phenyl)-2-methylbutyn-1,2-diol (14c, SPA7013)

Yellow solid (82%): *R*_f_ = 0.13 (CHCl_3_:EtOAc = 1:3); ^1^H NMR (500 MHz, CD_3_OD) δ 8.26 (s, 1H), 8.21 (s, 1H), 8.09 (d, *J* = 8.0 Hz, 1H), 7.48 (dd, *J* = 8.0, 7.8 Hz, 1H), 7.40 (d, *J* = 7.8 Hz, 1H), 3.63 (s, 2H), 2.69 (s, 3H), 1.56 (s, 3H); ^13 ^C NMR (125 MHz, CD_3_OD) δ 156.1, 143.5, 138.8, 129.0, 128.8, 123.9, 123.8, 121.0, 92.2, 82.4, 69.7, 68.2, 60.1, 24.8, 19.4, 13.1; HRMS m/z: 324.1471 [M + H]^+^; calculated C_17_H_18_N_5_O_2_^+^: 324.1461.

##### 2.1.2.21. 4–(3-(4-Amino-3-methylpyrazolo[3,4-*d*]pyrimidin-1-yl)phenyl)-2-phenylbutyn-2-ol (14d, SPA7014)

Pale yellow solid (46%): *R*_f_ = 0.33 (CHCl_3_:EtOAc = 1:5); ^1^H NMR (500 MHz, CD_3_OD) δ 8.26 (s, 1H), 8.24 (t, *J* = 1.6 Hz, 1H), 8.12 (dd, *J* = 8.2, 1.0 Hz, 1H), 7.76–7.71 (m, 2H), 7.51 (t, *J* = 7.9 Hz, 1H), 7.46–7.37 (m, 3H), 7.31 (d, *J* = 7.4 Hz, 1H), 2.70 (s, 3H), 1.85 (s, 3H); ^13 ^C NMR (125 MHz, CD_3_OD) δ 158.9, 156.1, 154.1, 146.0, 143.6, 138.9, 128.9, 128.8, 127.8, 127.1, 124.7, 123.8, 123.7, 121.1, 100.5, 93.6, 83.0, 69.3, 32.5, 13.1 HRMS m/z: 370.1679 [M + H]^+^; calculated C_22_H_20_N_5_O ^+^: 370.1668.

##### 2.1.2.22. General procedures for the synthesis of compounds 15a–b

*Step 1.* To a mixture of methanesulfonyl chloride (1.2 equiv.) in DCM, was slowly added propargyl alcohol (1.0 equiv.) and triethylamine (1.2 equiv.), simultaneously. The reaction mixture was stirred at room temperature for 1 h. The reaction mixture was washed with water and dried over MgSO_4_, filtered, and concentrated to provide methanesulfonic acid prop-2-ynyl ester as a yellow liquid without further purification. *Step 2.* To a mixture of methanesulfonic acid prop-2-ynyl ester in DCM, was added morpholine or piperidine (2.0 equiv.). After stirring at room temperature for 4 to 12 h, the reaction mixture was filtered. The combined filtrate was washed with NaHCO_3_ (aq) and dried over MgSO_4_, filtered, and concentrated *in vacuo*. The crude intermediates were used for the next step without further purification. *Step 3.* To a mixture of 3-Iodo-*1H*-pyrazolo[3,4-*d*]pyrimidin-4-amine (1.0 equiv.) and copper(I) iodide (0.2 equiv.) in DMF, was added tetrakis (triphenylphosphine)palladium(0) (0.1 equiv.) under a nitrogen atmosphere. After stirring at room temperature for 10 min, triethylamine (2.0 equiv.) and propargyl morpholine or propargyl piperidine (10.0 equiv.) were added. The reaction mixture was heated at 70 °C for 1.5 h. After cooling to room temperature, the mixture was filtered through a celite pad and rinsed with EtOAc. Water was added to the filtrate and the mixture was extracted with EtOAc. The combined organic layers were dried over MgSO_4_, filtered, and concentrated *in vacuo*. The residue was purified by flash column chromatography (EtOAc: MeOH) to provide compound **15**.

##### 2.1.2.23. 3–(3-Morpholin-4-ylprop-1-ynyl)-1*H*-pyrazolo[3,4-*d*]pyrimidin-4-ylamine (15a)

Pale yellow solid (47%): *R*_f_ = 0.22 (DCM:MeOH = 6:1); ^1^H NMR (500 MHz, CD_3_OD) δ 8.10 (s, 1H), 3.72–3.60 (m, 4H), 3.54 (s, 2H), 2.59 (br s, 4H).

##### 2.1.2.24. 3–(3-Piperidin-1-ylprop-1-ynyl)-1*H*-pyrazolo[3,4-*d*]pyrimidin-4-ylamine (15 b)

Pale yellow solid (47%): *R*_f_ = 0.22 (EtOAc:MeOH = 4:1); ^1^H NMR (500 MHz, CD_3_OD) δ 8.21 (s, 1H), 3.61 (s, 2H), 2.68 (br s, 4H), 1.74–1.65 (m, 4H), 1.53 (br s, 2H).

##### 2.1.2.25. General procedures for the synthesis of compounds 16a–b

To a mixture of compound **15** (1.0 equiv.) in MeOH, Pd/C (50 wt%) was added and the reaction mixture was then stirred within a hydrogen atmosphere for 1.5 h. The mixture was filtered through a celite pad, rinsed with MeOH, and concentrated *in vacuo*. The residue was purified by flash column chromatography (EtOAc:MeOH) to provide compound **16**.

##### 2.1.2.26. 3–(3-Morpholin-4-ylpropyl)-1*H*-pyrazolo[3,4-*d*]pyrimidin-4-ylamine (16a)

White solid (61%): *R*_f_ = 0.15 (EtOAc:MeOH = 4:1); ^1^H NMR (500 MHz, CD_3_OD) δ 8.18 (s, 1H), 3.70 (t, *J* = 4.6 Hz, 4H), 3.03 (t, *J* = 7.1 Hz, 2H), 2.53–2.40 (m, 6H), 1.98 (quint., *J* = 7.1 Hz, 2H).

##### 2.1.2.27. 3–(3-Piperidin-1-ylpropyl)-1*H*-pyrazolo[3,4-*d*]pyrimidin-4-ylamine (16 b)

Yellow solid (97%): *R*_f_ = 0.04 (EtOAc:MeOH = 4:1); ^1^H NMR (500 MHz, CD_3_OD) δ 8.16 (s, 1H), 3.02 (t, *J* = 7.2 Hz, 2H), 2.58–2.27 (m, 6H), 1.98 (quint., *J* = 7.2 Hz, 2H), 1.68–1.59 (m, 4H), 1.50 (br s, 2H).

##### 2.1.2.28. 1–(3-Bromophenyl)-3–(3-morpholin-4-ylpropyl)-1*H*-pyrazolo[3,4-*d*]pyrimidin-4-ylamine (17a)

White solid (57%): *R*_f_ = 0.30 (EtOAc:MeOH = 4:1); ^1^H NMR (500 MHz, CD_3_OD) δ 8.30 (t, *J* = 1.9 Hz, 1H), 8.18 (s, 1H), 8.09 (dt, *J* = 8.1, 1.9 Hz, 1H), 7.37–7.29 (m, 2H), 3.61 (t, *J* = 5.0 Hz, 4H), 3.00 (t, *J* = 7.1 Hz, 2H), 2.50–2.38 (m, 6H), 1.98 (quint., *J* = 7.1 Hz, 2H).

##### 2.1.2.29. 1–(3-Bromophenyl)-3–(3-piperidin-1-ylpropyl)-1*H*-pyrazolo[3,4-*d*]pyrimidin-4-ylamine (17 b)

White solid (52%): *R*_f_ = 0.14 (EtOAc:MeOH = 4:1); ^1^H NMR (500 MHz, CD_3_OD) δ 8.43 (t, *J* = 1.9 Hz, 1H), 8.30 (s, 1H), 8.24–8.20 (m, 1H), 7.51–7.42 (m, 2H), 3.10 (t, *J* = 7.2 Hz, 2H), 2.59–2.44 (m, 6H), 2.09 (quint., *J* = 7.2 Hz, 2H), 1.70–1.61 (m, 4H), 1.52 (br s, 2H).

##### 2.1.2.30. (3–(4-Amino-3–(3-morpholin-4-ylpropyl)pyrazolo[3,4-*d*]pyrimidin-1-yl)phenylethynyl)cyclohexanol (18a, SPA7016)

Off-white solid (66%): *R*_f_ = 0.33 (EtOAc:MeOH = 4:1); ^1^H NMR (500 MHz, CD_3_OD) δ 8.28 (s, 1H), 8.20 (t, *J* = 2.0 Hz, 1H), 8.14 (ddd, *J* = 8.0, 2.0, 1.0 Hz, 1H), 7.50 (t, *J* = 8.0 Hz, 1H), 7.38 (dt, *J* = 8.0, 1.0 Hz, 1H), 3.72 (t, *J* = 4.6 Hz, 4H), 3.12 (t, *J* = 7.1 Hz, 2H), 2.58–2.43 (m, 6H), 2.13–1.98 (m, 4H), 1.83–1.59 (m, 7H), 1.35 (br s, 1H); ^13 ^C NMR (125 MHz, CD_3_OD) δ 156.0, 154.1, 146.9, 138.9, 128.8, 124.0, 123.7, 120.8, 100.4, 93.7, 82.9, 68.0, 66.2, 57.0, 53.3, 39.5, 39.0, 25.2, 25.0, 24.9, 23.0, 13.1; HRMS m/z: 461.2666 [M + H]^+^; calculated C_26_H_33_N_6_O_2_^+^: 461.2665.

##### 2.1.2.31. (3-[4-Amino-3–(3-piperidin-1-ylpropyl)pyrazolo[3,4-*d*]pyrimidin-1-yl]phenylethynyl)cyclohexanol (18 b, SPA7017)

Off-white solid (40%): *R*_f_ = 0.36 (EtOAc:MeOH = 4:1); ^1^H NMR (500 MHz, CD_3_OD) δ 8.22 (s, 1H), 8.17 (s, 1H), 8.10 (d, *J* = 8.0 Hz, 1H), 7.43 (dd, *J* = 8.0, 7.6 Hz, 1H), 7.32 (d, *J* = 7.6 Hz, 1H), 3.00 (t, *J* = 7.0 Hz, 2H), 2.48–2.30 (m, 5H), 2.04–1.93 (m, 4H), 1.80–1.37 (m, 14H), 1.28 (s, 1H); HRMS m/z: 459.2875 [M + H]^+^; calculated C_27_H_35_N_6_O^+^: 459.2872.

### 2.2. Kinase activity assay

To examine the enzyme activity, HotSpot kinase assay was performed at Reaction Biology Corporation (Malvern, PA, USA). The kinase and substrate pairs were prepared in reaction buffer: 20 mM HEPES at pH 7.5, 10 mM MgCl_2_, 1 mM EGTA, 0.02% Brij 35, 0.02 mg/mL BSA, 0.1 mM Na_3_VO_4_, 2 mM DTT, and 1% DMSO. Compounds were added into the kinase reaction mixture and incubated for 20 min. Then, ^32 ^P-ATP was introduced to the mixture to initiate the reaction carried out at 10 μM of ATP concentration. After 2 h at room temperature, the mixture was spotted onto P81 ion exchange paper and kinase activity was then evaluated utilising the filter-binding method. Compounds were tested for single dose enzyme inhibition at a concentration of 20 μM. For the control compound, staurosporine was tested in a 10-dose IC_50_ mode. To determine the IC_50_ value, the compounds were tested for 10-doses with 3-fold serial dilution starting at 100 μM.

### 2.3. Molecular docking analysis

Molecular docking was carried out using Cresset Flare V.5.0 (Litlington, Cambridgeshire, UK). 3 D coordinates of the active site were taken from the crystal structure of the human PAK4 in complex with ligands (PDB code 4O0V) from the RCSB Protein Data Bank. The protein structure was prepared and minimised, and the grid box of the binding site was defined according to the clustered ligand. The docking calculations of synthesised compounds were carried out using Flare V.5.0 in normal docking mode.

### 2.4. Model of partial hepatic I/R injury

Pathogen-free 8 to 10-week old C57BL/6 male mice (Orient Bio, Seoul, Korea) were kept under a 12-h light/dark cycle with controlled humidity of 50% at 22 °C, and had free access to food and water. Hepatic I/R injury was created using the following method.^15^ Mice were anaesthetised with ketamine (100 mg/kg) and xylazine (10 mg/kg) by intraperitoneal injection. A midline incision was performed, and an atraumatic clip was placed across the portal vein, hepatic artery, and bile duct just above the right branch. Blood flow was interrupted to the left lateral and median lobes, which represent approximately 70% of the total blood supply to the liver. The liver was kept moist with a gauze soaked in saline, and body temperature was maintained at 37 °C with a warm blanket throughout ischaemia. After 60 min of partial hepatic ischaemia, the clip was removed to initiate reperfusion. The sham mice underwent the same operation without vascular occlusion. After the desired time period of reperfusion, the mice were euthanised by exsanguination under anaesthesia and serum samples were collected. The left lateral and median lobes of the liver were collected and stored at −80 °C until further analysis, or were immediately fixed in 10% formalin. All animal experiments were performed in accordance with the Guide for the Care and Use of Laboratory Animals published by the US National Institutes of Health (NIH Publication No. 85–23, revised 2011). The study protocol was approved by the Institutional Animal Care and Use Committee of Jeonbuk National University (permit number: JBNU-2019–00122).

### 2.5. Isolation of primary hepatocytes

Primary hepatocytes were prepared from 8 to 10 week old C57BL/6 male mice by perfusion. After intubation in the inferior vena cava, the liver was perfused with a calcium-free HEPES buffer (10 mM, pH 7.4) for 5 min at a flow rate of 0.35 mL/min, followed by HEPES buffer containing 5 μg of collagenase IV (Sigma-Aldrich, St. Louis, MO, USA) with 5 mM calcium chloride for 5 min. Hepatocytes were resuspended in DMEM/F12 supplemented with 10% FBS, 10 units/mL penicillin, 10 μg/mL streptomycin, and 10 nM dexamethasone mixed with 42% Percoll, and then centrifuged for 5 min at 1,300 rpm. The cells were plated at 1 × 10^6^ cells/well in 6-well culture dishes.

### 2.6. Hypoxia-reoxygenation protocol

Primary hepatocytes were incubated at 37 °C in anaerobic jars (Oxoid, Basingstoke, Hampshire, UK) with oxygen-absorbing packs (AnaeroGen, Oxoid). This method has been shown to achieve controlled oxygen levels below 1%. Following various periods of hypoxia, reoxygenation of cells was initiated by opening the chamber and replacing the hypoxic medium with an oxygenated medium.

### 2.7. Cell culture and transient transfection

The human embryonic kidney cell line HEK293T was obtained from the American Type Culture Collection (Manassas, VA, USA). For the Nrf2 reporter gene assay, 1 μg of a plasmid containing a promoter with an ARE driving luciferase expression was used. Exogenous proteins were expressed by transfecting HEK293T cells with 1 μg of Nrf2 and PAK4 with Lipofectamine 3000 (Invitrogen, Carlsbad, CA, USA). Luciferase activity was measured using a Dual-Luciferase Reporter Assay (Promega, Madison, WI, USA) by Lumat LB 9507 (Berthold, Bad Wildbad, Germany).

### 2.8. MTT assay for cell viability

Cell viability was determined by analysing the reduction of 3–(4, 5-dimethylthiazol-2-yl)-2, 5-diphenyltetrazolium bromide (MTT) to formazan.

### 2.9. Flow cytometry analysis of apoptosis

H/R-induced apoptosis was monitored using the Annexin V-propidium iodide (PI) double-staining kit (BD Biosciences, San Jose, CA, USA) according to the instructions of the manufacturer, allowing quantification by flow cytometry (BD Biosciences). The proportion of apoptotic cells was defined as the percentage of Annexin V-positive cells relative to PI-positive cells.

### 2.10. Measurement of cellular ROS levels

Intracellular ROS levels were measured using 2′,7′-dichlorofluorescein diacetate (DCF-DA), which converts to fluorescent DCF-DA in cells when exposed to ROS. Briefly, after H/R injury, hepatocytes were loaded with 20 μM DCF-DA (Invitrogen) for 10 min at 37 °C. The DCF fluorescence intensity was measured by fluorescence spectrophotometry with 485 nm excitation and 538 nm emission and expressed as a percentage relative to control.

### 2.11. Biochemical analysis

Alanine aminotransferase (ALT) and aspartate aminotransferase (AST) in serum (Asan Pharm, Seoul, Korea), glutathione (GSH, Arbour Assays, Ann Arbour, MI, USA), malondialdehyde (MDA, ab118970, Abcam, Cambridge, UK) in liver tissues, and lactate dehydrogenase (LDH, Biovision, Milpitas, CA, USA) in culture medium were analysed using specific kits. Serum levels of IL-1β, IL-6, TNF-α, and CCL-2 were detected using ELISA kits (all from Invitrogen).

### 2.12. RNA isolation and qPCR

Total RNA was extracted from frozen liver tissues or primary hepatocytes using TRIzol reagent (Invitrogen). RNA was treated with isopropanol, dried using 70% ethanol, and dissolved in diethyl pyrocarbonate-treated distilled water. First-strand cDNA was generated using the random hexamer primer provided in the first-strand cDNA synthesis kit (Applied Biosystems, Foster City, CA, USA). Specific primers were designed using PrimerBank (https://pga.mgh.harvard.edu/primerbank, Table 1). qPCR reactions were performed in a final volume of 10μ L containing 10 ng reverse-transcribed total RNA, 200 nM forward and reverse primers, and PCR master mixture. qPCR was performed in 384-well plates using an ABI Prism™ 7900HT Sequence Detection System (Applied Biosystems).

**Table 1.** Sequences and accession numbers for primers (forward, FOR; reverse, REV) used in qPCR

Gene	Sequences for primers	Accession no.
*Tnfa*	FOR: AGGGTCTGGGCCATAGAACT	NM_013693
	REV: CCACCACGCTCTTCTGTCTAC	
*Il1b*	FOR: GGTCAAAGGTTTGGAAGCAG	NM_008361
	REV: TGTGAAATGCCACCTTTTGA	
*Il6*	FOR: CCACGGCCTTCCCTACTTC	NM_031168
	REV: TTGGGAGTGGTATCCTCTGTGA	
*Icam1*	FOR: AACAGTTCACCTGCACGGAC	NM_010493
	REV: GTCACCGTTGTGATCCCTG	
*Cxcl1*	FOR: AATGAGCTGCGCTGTCAGTG	NM_008176
	REV: TGAGGGCAACACCTTCAAGC	
*Ccl2*	FOR: ACCGACAACAGGAAGTGGAG	NM_009140
	REV: TGGACGTTTCACACAGTGGT	
*Ho1*	FOR: AGGTACACATCCAAGCCGAGA	NM_010442
	REV: CATCACCAGCTTAAAGCCTTCT	
*Nqo1*	FOR: AGGATGGGAGGTACTCGAATC	NM_008706
	REV: TGCTAGAGATGACTCGGAAGG	
*Nrf2*	FOR: CTTTAGTCAGCGACAGAAGGAC	NM_010902
	REV: AGGCATCTTGTTTGGGAATGTG	
*Gapdh*	FOR: CGTCCCGTAGACAAAATGGT	NM_008084
	REV: TTGATGGCAACAATCTCCAC	

### 2.13. Subcellular fractionation, Western blotting, and co-immunoprecipitation (Co-IP)

Nuclear and cytoplasmic fractions were prepared using the NE-PER Nuclear and Cytoplasmic Extraction kit (Thermo Fisher Scientific, Waltham, MA, USA). Tissue homogenates or cell lysates (15 μg) were separated by 6–14% SDS-PAGE and transferred to PVDF membranes. After blocking with 5% skim milk, blots were probed with primary antibodies against: PAK4 (G222), p-PAK4 (S474), p-IKKβ (S176/180), p-IκBα (S32), p-p65 (S536), cleaved caspase-3 (Asp175), Bax (Cell Signalling Technology, Beverly, MA, USA), GAPDH (A531), lamin B1(L75), Bcl2(P65), NQO1 (F252) (Bioworld Technology, St Louis Park, MN, USA), NF-κB p65 (C-20), IKBα (H-4), IKKβ (10A9B6) (Santa Cruz Biotechnology, Dallas, TX, USA), Nrf2 (Proteintech, Rosemont, IL, USA), HO-1 (Enzo Life Sciences, Farmingdale, New York, USA), and p-Thr (Merck KGaA, Darmstadt, Germany). For co-immunoprecipitations, 600 μg protein was incubated with anti-Nrf2 antibodies (Proteintech, Sankt Leon-Rot, Germany) overnight at 4 °C followed by protein G agarose for 2 h at 4 °C. Blots were probed with antibodies against PAK4, Nrf2, or p-Thr. Immunoreactive bands were detected with a Las-4000 imager (GE Healthcare Life Science, Pittsburgh, PA, USA).

### 2.14. Histology

Paraffin sections of liver tissue (5 μm) were stained with haematoxylin and eosin (H&E) for light microscopy. Five random sections were investigated per slide to measure the necrotic area and determine the percentage of apoptotic cells. To measure the necrotic areas, sections were observed under an Axiovert 40 CFL microscope (Carl Zeiss, Oberkochen, Germany) and measured using iSolution DT 36 software (Carl Zeiss). For immunofluorescence staining, after deparaffinisation, sections were immunostained with antibodies against F4/80 (ab6640), Ly6G (ab25377), and 4-hydroxynonenal (4-HNE, ab46545) (all from Abcam). TUNEL staining was performed with commercial kits (Promega). After washing with phosphate buffered saline (PBS), secondary antibodies (Alexa Fluor 488-conjugated goat anti-mouse IgG1 and Alexa Fluor 594-conjugated goat anti-rabbit IgM, Thermo Fisher Scientific) were incubated for 1 h at 37 °C. Sections were counterstained with DAPI.

### 2.15. Statistical analysis

Data are expressed as the mean ± standard deviation of the mean (SD). Statistical comparisons were made using one-way analysis of variance followed by Tukey’s *post hoc* analysis. The significance of differences between the two groups was determined using Student’s unpaired *t*-test. A *p* values of less than 0.05 was considered significant. All analyses were performed using GraphPad Prism 9.3 (San Diego, CA, USA).

## 3. Results

### 3.1. Chemistry

**Figure 1.**
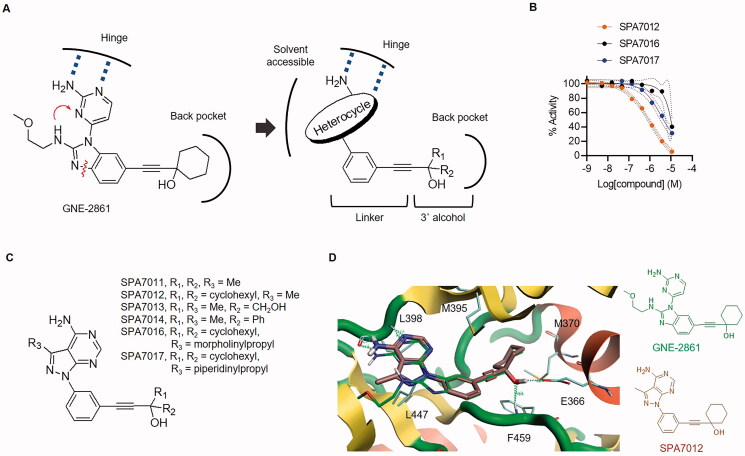
**Drug design and identification of SPA7012 as PAK4 inhibitor.** (A) Design strategy for novel PAK4 selective inhibitors. (B) Enzymatic IC_50_ curves for SPA7012, SPA7016 and SPA7017. (C) Representative structure of pyrido[3,4-*d*]pyrimidine derivatives. (D) Predicted binding mode of SPA7012 (PDB code: 4O0V). Original ligand GNE-2861 (green) and SPA7012 (brown) are presented in the stick model. The hydrogen bonds are indicated as a green dashed line.

Structure-based drug design was performed to identify novel PAK4 selective inhibitors. GNE-2861 is regarded as the most selective and potent PAK4 inhibitor targeting the specific back pocket of PAK4 caused by abnormal R-spine rearrangement.^16^^,^^17^ Therefore, we adopted GNE-2861 as a lead for drug design. To find new selective PAK4 inhibitors with improved properties, attempts were made to change the adenine pocket-binding group while maintaining the back pocket targeting group of GNE (Figure 1(A)). Scaffold hopping of adenine mimic monocycle to bicycle or tricycle was performed, expecting an improved log *p* values and easy-to-add solvent-accessible moieties.^18^ Following these changes in the adenine pocket-binding group, the bicyclic linker turned to a monocycle to reduce the structural rigidity of the entire compound. *In silico* fragment screening provided heterocyclic scaffolds with structural novelty. Two privileged structures, namely, pyrido[4,3-*b*]indole (used as a synonym of γ-carboline) and pyrazolo[3,4-*d*]pyrimidine, were selected and designed as type I ½ inhibitors.

**Scheme 1.**
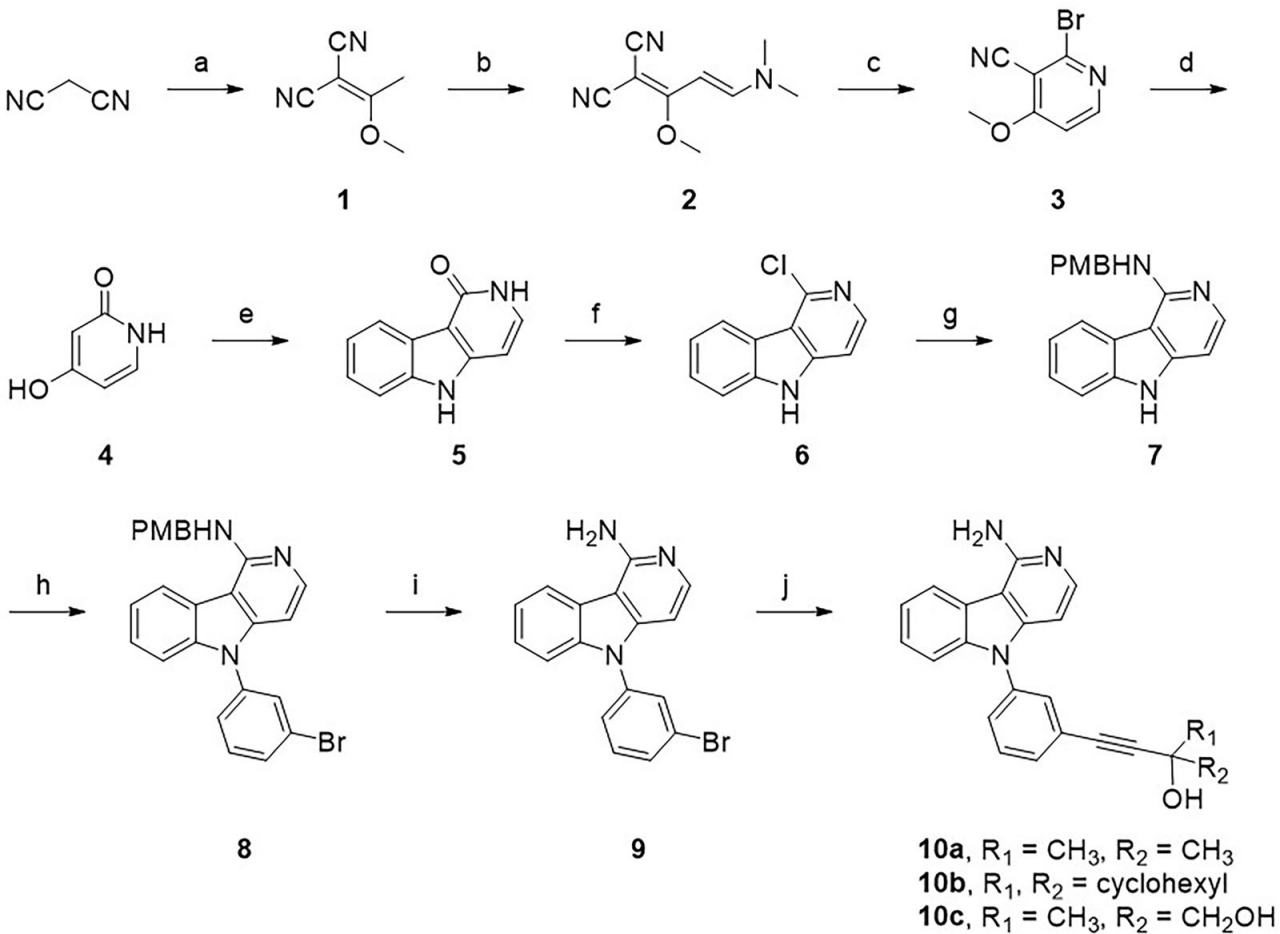
**Synthesis of 1-aminopyrido[4,3-*b*]indole derivatives.** Reagents and conditions: (a) trimethyl orthoacetate, 100 °C; (b) DMF-DMA, MeOH, reflux; (c) 48% HBr, AcOH, room temperature; (d) conc. HCl, 160 °C; (e) phenylhydrazine, Ph_2_O, 240 °C; (f) POCl_3_, reflux; (g) *p*-methoxybenzylamine, 200 °C; (h) 1-bromo-3-iodobenzene, Cu, K_2_CO_3_, 18-crown-6, 1,2-dichlorobenzene, 180 °C; (i) TFA, 60 °C; and (j) *R*-prop-2-yn-1-ol, Pd(PPh_3_)_2_Cl_2_, TEA/DMSO (2:1), 70 °C.

**Scheme 2.**
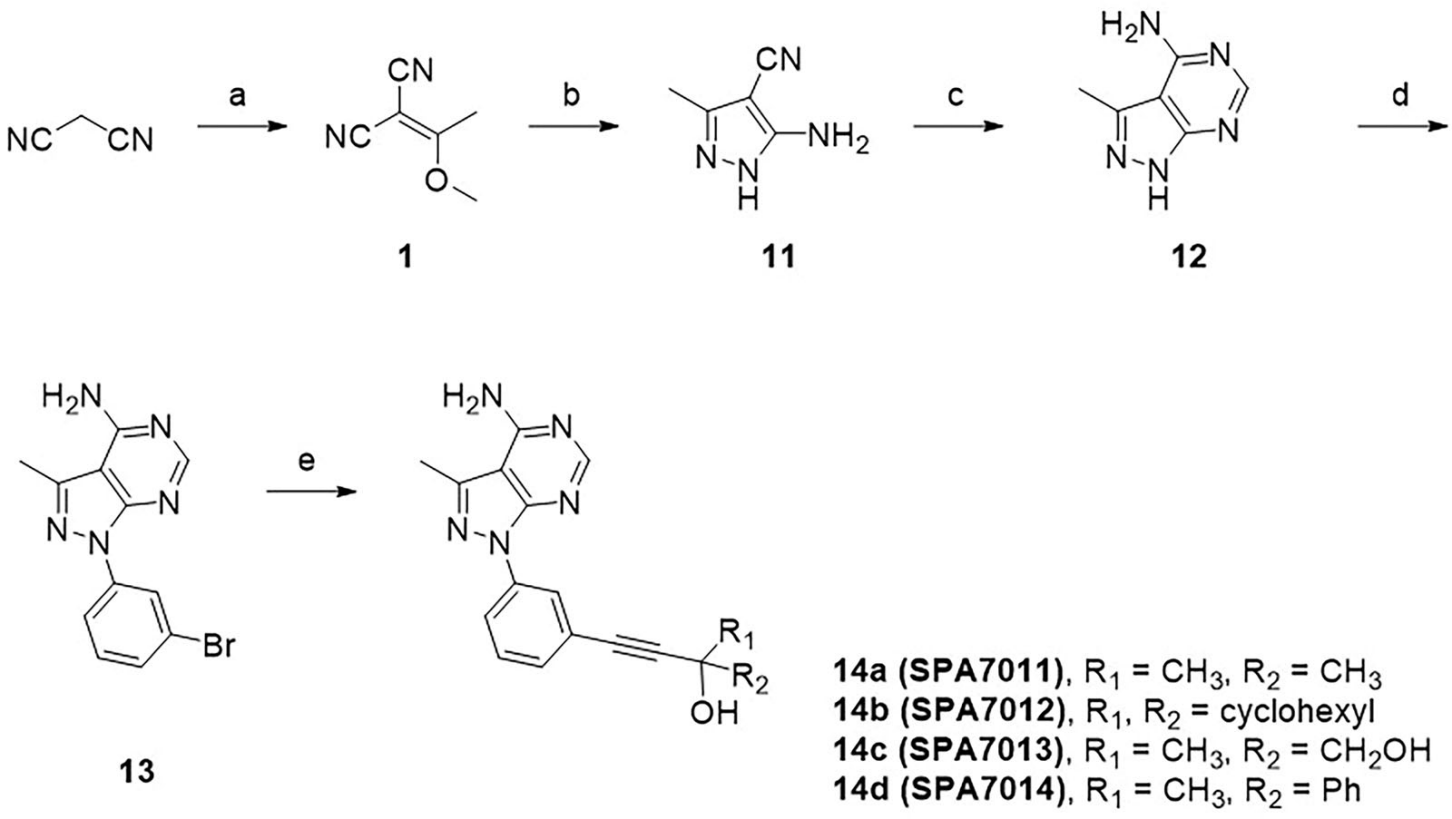
**Synthesis of the 4-aminopyrazolo[3,4-*d*]pyrimidine derivatives.** Reagents and conditions: (a) trimethyl orthoacetate, 100 °C; (b) hydrazine monohydrate, EtOH, 80 °C; (c) formamide, 180 °C; (d) 1-bromo-3-iodobenzene, CuI, K_2_CO_3_, DMEDA, DMF, 110 °C; and (e) R-prop-2-yn-1-ol, Pd(PPh_3_)_2_Cl_2_, TEA/DMSO (2:1), 70 °C.

**Scheme 3.**
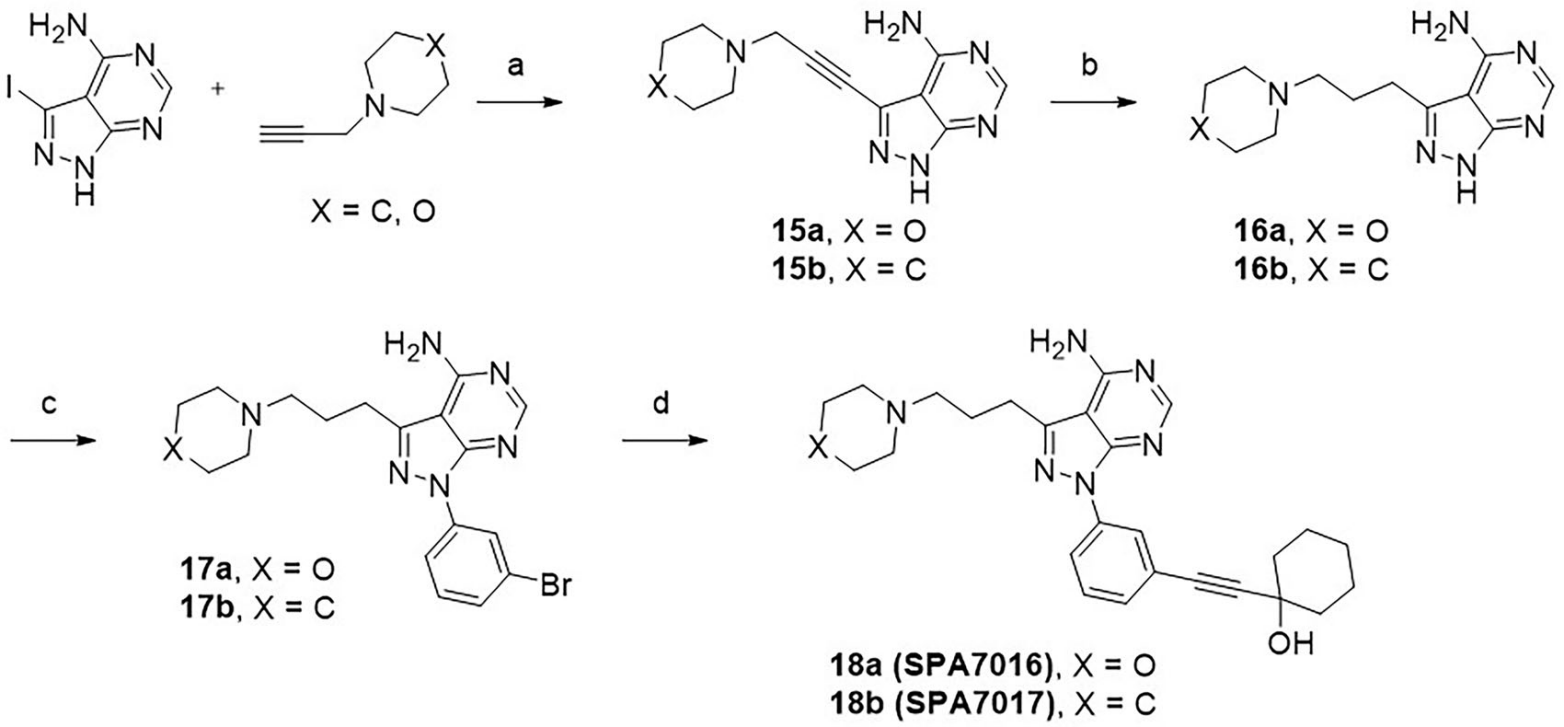
**Synthesis of the 4-aminopyrazolo[3,4-*d*]pyrimidine derivatives with a solvent-accessible moiety**. Reagents and conditions: (a) CuI, Pd(PPh_3_)_4_, TEA, DMF, 70 °C; (b) Pd/C, H_2_, MeOH, room temperature; (c) 1-bromo-3-iodobenzene, CuI, K_2_CO_3_, DMEDA, DMF, 110 °C; and (d) 1-Ethynylcyclohexanol, Pd(PPh_3_)_2_Cl_2_, TEA/DMSO (2:1), 70 °C.

A series of pyrido[4,3-*b*]indole derivatives were synthesised, as depicted in Scheme 1. Knoevenagel condensation of malononitrile with trimethyl orthoacetate yielded the enol ether.^1^ Enamine^2^ was obtained by treatment with *N,N*-dimethylformamide dimethyl acetal (DMF-DMA) and was converted to pyridine^4^ under acidic conditions. Fischer indole synthesis between 2,4-dihydroxypyridine^4^ and phenylhydrazine yielded a pyrido[4,3-*b*]indole moiety.^5^ The carbonyl group of pyridone^5^ was converted to chloropyridine^6^ by treatment with phosphoryl chloride, and the resulting chloropyridine was substituted with a *p*-methoxybenzyl (PMB) protected amine, which yielded aminopyridine.^7^ The Ullmann-type reaction of 5*H*-pyrido[4,3-*b*]indole with 1-bromo-3-iodobenzene resulted in *N*-phenyl-substituted pyridoindole,^8^ and deprotection of the PMB protective amine yield the free amine.^9^ The lipophilic groups targeting the back pocket were introduced by Sonogashira coupling between aryl halide^9^ and alkynes, which produced the target compound **10**. Pyrazolo[3,4-*d*]pyrimidine derivatives were also synthesised as depicted in Scheme 2 and Scheme 3. The enol ether^1^ synthesised from malononitrile was treated with hydrazine to obtain an aminopyrazole ring,^11^ and subsequent cyclisation with formamide provided a 4-aminopyrazolo[3,4-*d*]pyrimidine scaffold.^12^ Subsequent Ullmann reaction and Sonogashira coupling reaction provided the target compound **14**. A solvent-accessible moiety was then introduced to the 4-aminopyrazolo[3,4-*d*]pyrimidine backbone. Sonogashira coupling of 3-iodopyrazolo[3,4-*d*]pyrimidine with a substituted propyne introduced a solvent-accessible moiety, and hydrogenation yielded compound **16**. Similar to the previous compounds, Ullmann-type coupling and Sonogashira coupling reactions were performed sequentially to obtain the target compound **18**.

Synthesised compounds were evaluated for their *in vitro* PAK4 enzyme inhibitory activities. *In vitro* kinase assays were performed using HotSpot kinase assays for relative enzyme inhibitory activity at a single concentration (20 μM) and IC_50_ assessment at an ATP concentration of 10 μM (Figure 1(B)). The bicyclic pyrazolo[3,4-*d*]pyrimidine derivative (Figure 1(C)) showed increased activity than the tricyclic pyrido[4,3-*b*]indole derivative (Table 2). For the allosteric pocket binder, cyclohexyl group (SPA7012) was preferable to the smaller dimethyl (SPA7011) or hydroxymethyl group (SPA7013), and the aromatic ring (SPA7014) reduced activity compared to the saturated ring (SPA7012). *In vitro* enzyme IC_50_ values appeared in the order of SPA7012, SPA7017, and SPA7016. Compound SPA7012 with a submicromolar IC_50_ value was selected to evaluate subtype selectivity (Table 3), and showed enhanced selectivity for PAK4 (81% inhibition, 0.77 μM of IC_50_) compared to PAK1 (16% inhibition) and PAK2 (36% inhibition, >100 μM of IC_50_). Molecular docking analysis was performed on the human PAK4 catalytic domain structure complexed with GNE-2861 (PDB code: 4O0V). The binding pose of SPA7012 overlapped well with the crystal structure of GNE-2861, with the allosteric binder properly positioned in the specific back pocket. The oxygen atom in the tertiary alcohol of the hydrophobic allosteric binder formed hydrogen bonds with the backbone of Phe459 and the side chain of Glu366 (Figure 1(D)). The amine group and nitrogen atom in the pyrazolo[3,4-*d*]pyrimidine core scaffold also formed hydrogen bonds with Lue398 at the hinge region. Finally, SPA7012 was decided as a lead compound for further examination with satisfactory potency and subtype selectivity.

**Table 2.** PAK4 enzyme inhibitory activity and calculated properties of synthesised compounds

	PAK4 enzyme inhibition		
Compound	% inhibitory activity	IC_50_ (μM)^b^	clogP
10a	32	ND	3.79
10b	54	34.0	5.10
10c	29	ND	2.66
14a	60	ND	1.25
14b	97	0.77	2.57
14c	36	ND	0.12
14d	65	ND	2.59
18a	88	9.92	2.57
18b	90	4.41	3.78

ND: not determined

^a^Relative percentage inhibition at 20 μM of compounds with staurosporine, HotSpot kinase assay (Reaction Biology Corp.).

^b^HotSpot kinase assay (Reaction Biology Corp.).

**Table 3.** PAK subtype selectivity of SPA7012

Subtype	% inhibitory activity^a^	IC_50_ (µM)^b^
PAK1	16	ND
PAK2	36	NA
PAK3	5	ND
PAK4	81	0.77
PAK5	52	>100
PAK6	36	NA

ND: not determined; NA: no activity

^a^Relative percentage inhibition at 20 μM of compounds with staurosporine, HotSpot kinase assay (Reaction Biology Corp.).

^b^HotSpot kinase assay (Reaction Biology Corp.).

### 3.2. Intraperitoneal administration of SPA7012 attenuates hepatic I/R injury in mice

**Figure 2.**
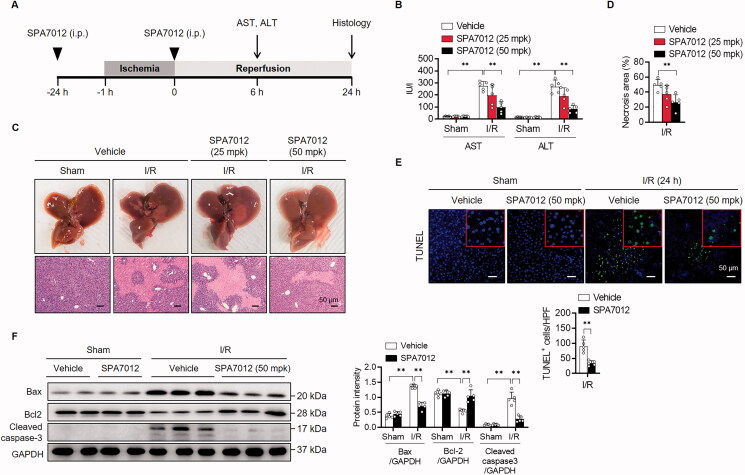
**Attenuation of hepatic I/R injury by SPA7012.** (**A**) Schematic diagram of treatment of SPA7012 in C57BL/6 mice. (**B**) Serum levels of AST and ALT (*n* = 5). (**C, D**) Gross morphology of livers, microscopic pictures of liver sections and measurement of necrotic area (*n* = 5). (**E**) Immunofluorescence staining and quantification of TUNEL-positive apoptotic cells in liver tissues (*n* = 5). (**F**) Western blotting analysis of apoptosis-related proteins in liver tissues (*n* = 5). Values are the mean ± SD.** *p* < 0.01. I/R, ischaemia-reperfusion; HPF, high power field.

We first examined whether SPA7012 has an effect on liver damage in mice subjected to 1 h of ischaemia and 6 h or 24 h of reperfusion (Figure 2(A)). Liver injury as assessed by serum levels of AST and ALT was evident in mice undergoing I/R compared to those in sham-operated mice (Figure 2(B)). Administration of SPA7012 before and during I/R injury at a dose of 50 mg/kg significantly decreased both AST and ALT levels in I/R injured mice. Gross findings of liver morphology and liver necrosis were consisted with AST and ALT levels (Figure 2(C)). H&E staining revealed extensive hepatocellular necrosis in I/R-injured liver tissues compared with the sham group (Figure 2(C,D)). However, in SPA7012 treated mice, the area of necrosis was significantly smaller compared with the I/R group and there were considerable areas of normal liver structure.

Although the major cause of cell death during hepatic I/R injury is necrosis, apoptotic cell death was also observed during the injury process.^19^^,^^20^ The number of TUNEL-positive apoptotic cells was significantly larger in I/R-injured liver tissues while TUNEL-positive cells were rarely observed in the sham group (Figure 2(E)). Western blotting analyses showed increases in pro-apoptotic Bax and cleaved caspase-3, and a decrease in anti-apoptotic Bcl-2 in I/R injured livers (Figure 2(F)). When mice were treated with SPA7012, a significant reduction in TUNEL-positive cells and concordant changes in apoptotic proteins were observed. Together, these results indicate that SPA7012 has protective effects against hepatocellular damage upon I/R injury.

### 3.3. SPA7012 reduces oxidative stress in mice with I/R injury

**Figure 3.**
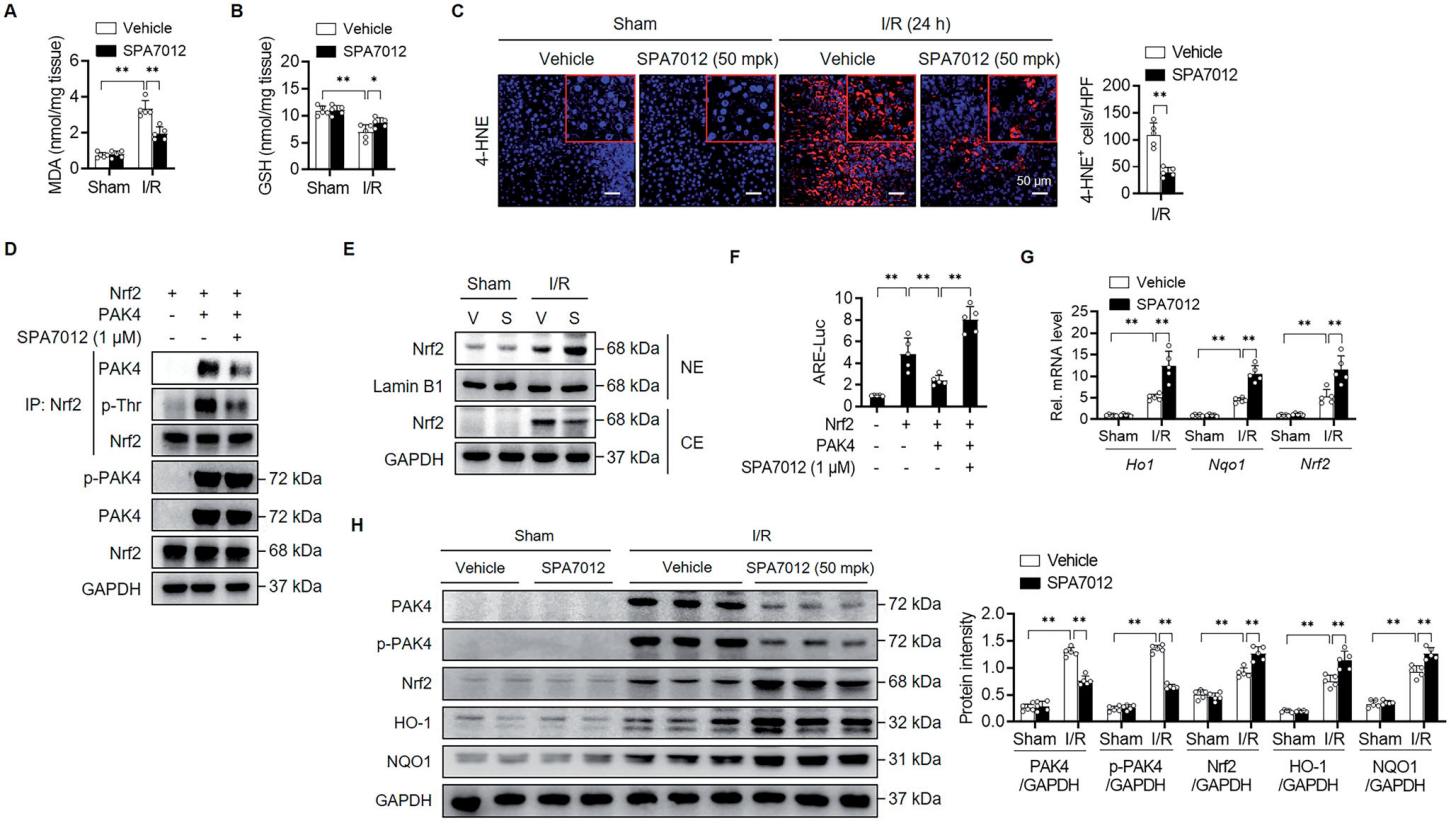
**Attenuation of I/R-induced oxidative stress by SPA7012.** (A and B) Hepatic levels of malondialdehyde (MDA) and glutathione (GSH) (*n* = 5). (C) Immunohistochemical staining for 4-hydroxynonenal (4-HNE) of liver tissues (*n* = 5). (D) A co-IP analysis in HEK293T cells after 24 h transfection. (E) Nrf2 protein levels in nuclear-(NE) and cytosolic extracts (CE) of I/R injured liver tissues. (F) ARE luciferase activity in HEK293T cells after 24 h transfection (*n* = 5). (G and H) Nrf2 and its target gene levels in liver tissues (*n* = 5). Values are the mean ± SD. **p < 0.01. I/R: ischaemia–reperfusion; HPF: high-power field; V: vehicle; S: SPA7012 50 mg/kg.

To determine whether SPA7012 provided protection against I/R injury, we measured oxidative stress markers in liver tissues. MDA levels (an indicator of lipid peroxidation) were greatly increased in I/R injured liver tissues compared with the sham group (Figure 3(A)). Conversely, I/R caused a significant suppression of antioxidant GSH levels in liver tissues (Figure 3(B)). Immunostaining of liver tissues with 4-HNE revealed an increase of lipid peroxidation subsequent to I/R injury (Figure 3(C)). However, SPA7012 treatment reversed changes caused by I/R; the serum level of MDA was significantly lower, hepatic levels of GSH were significantly higher, and 4-HNE-positive cells were significantly lower in the SPA7012 group than the I/R group.

Our previous study found that PAK4 phosphorylates Nrf2 at Thr369, which triggers its nuclear export and subsequent proteasomal degradation in the cytoplasm.^9^ Therefore we investigated whether SPA7012 could affect subcellular localisation, protein stability, and transcriptional activity of Nrf2. Co-IP analysis after the overexpression of Nrf2 and PAK4 in HEK293T cells showed that SPA7012 effectively inhibited PAK4-mediated phosphorylation of Nrf2 at threonine residue (Figure 3(D)). Consistently SPA7012 treatment increased the nuclear level of Nrf2 in I/R injured liver tissues (Figure 3(E)) and Nrf2 transcriptional activity in HEK293T cells (Figure 3(F)). qPCR and Western blotting analyses further confirmed the enhanced transcriptional activity of Nrf2 by SPA7012 treatment in I/R injured liver tissues (Figure 3(G,H)). These results indicate that SPA7012 exhibits hepatoprotective effects against I/R injury through stabilising Nrf2 protein and enhancing antioxidant protein expression.

### 3.4. SPA7012 suppresses inflammation in mice with I/R injury

**Figure 4.**
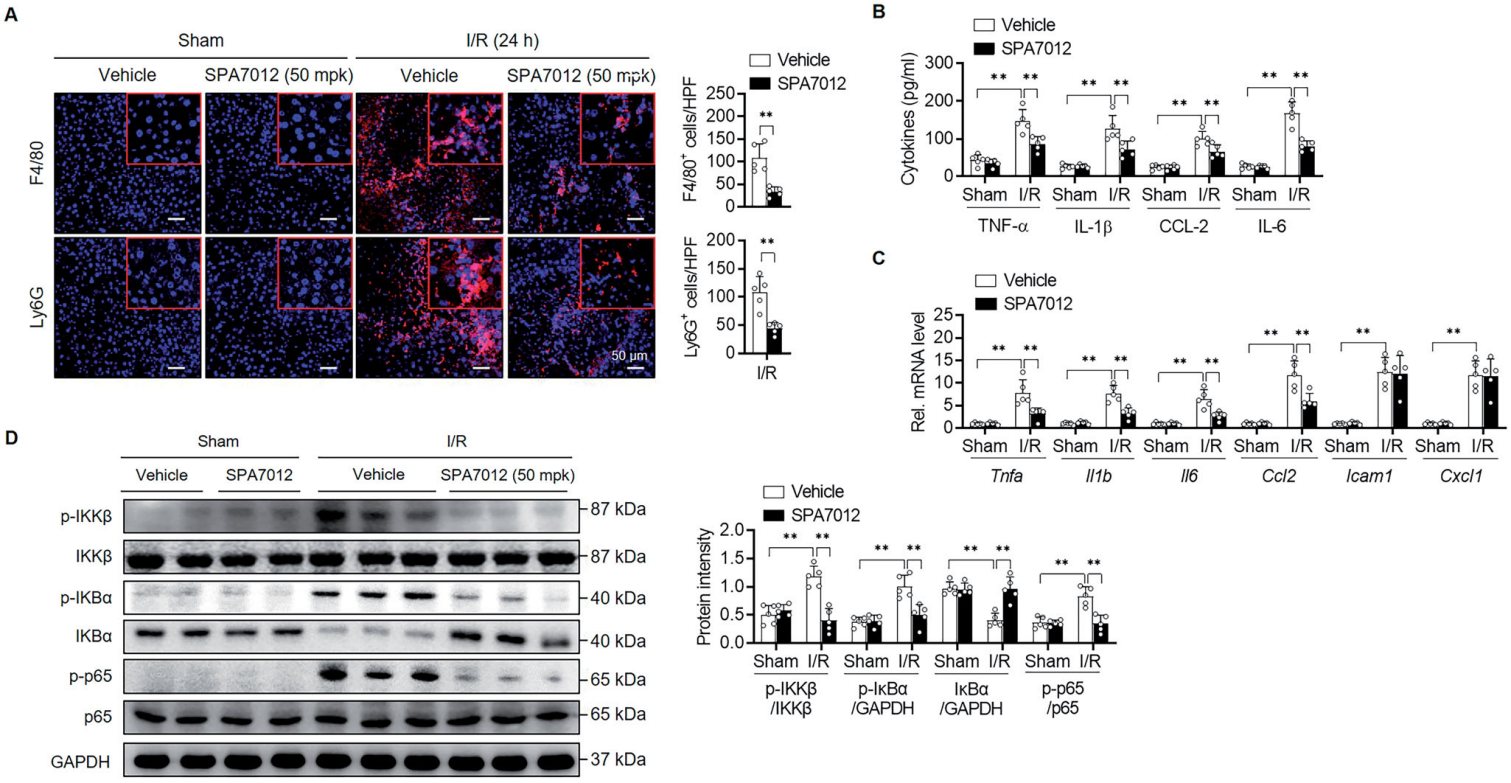
**Attenuation of I/R-induced inflammation by SPA7012.** (A) Immunofluorescence staining and quantification of F4/80-positive macrophages and Ly6G-positive neutrophils in liver tissues (*n* = 5). (B and C) The protein and mRNA levels of pro-inflammatory cytokines/chemokine in serum and liver tissues (*n* = 5). (D) Protein levels of NF-κB signalling pathway (*n* = 5). Values are the mean ± SD. **p < 0.01. HPF: high-power field.

Hepatic I/R is also characterised by sterile inflammation, in which resident Kupffer cells are activated or immune cells (monocytes and neutrophils) are recruited to the liver tissues in response to hepatocyte death. We first immunostained liver tissues to evaluate the degree of inflammation 24 h after reperfusion. The number of F4/80-positive macrophages and Ly6G-positive neutrophils were both notably increased during hepatic I/R (Figure 4(A)), indicating inflammation in the liver. The levels of proinflammatory cytokines/chemokines (TNF-α, IL-1β, IL-6, and CCL-2) in the serum and corresponding mRNA levels in the liver were also significantly increased by I/R injury (Figure 4(B,C)). However, the treatment of mice with SPA7012 decreased inflammatory cell infiltration and cytokine production. As NF-κB is closely correlated with inflammatory cytokine production, we analysed the NF-κB signalling pathway by Western blotting. We found that SPA7012 treatment substantially decreased phosphorylation levels of IKKβ and p65, and increased protein levels of IκBα compared to those of sham mice (Figure 4(D)). These results confirm that suppression of inflammation utilising SPA7012 may further provides to protection against I/R injury.

### 3.5. SPA7012 suppresses H/R-induced apoptotic cell death and inflammation in hepatocytes

**Figure 5.**
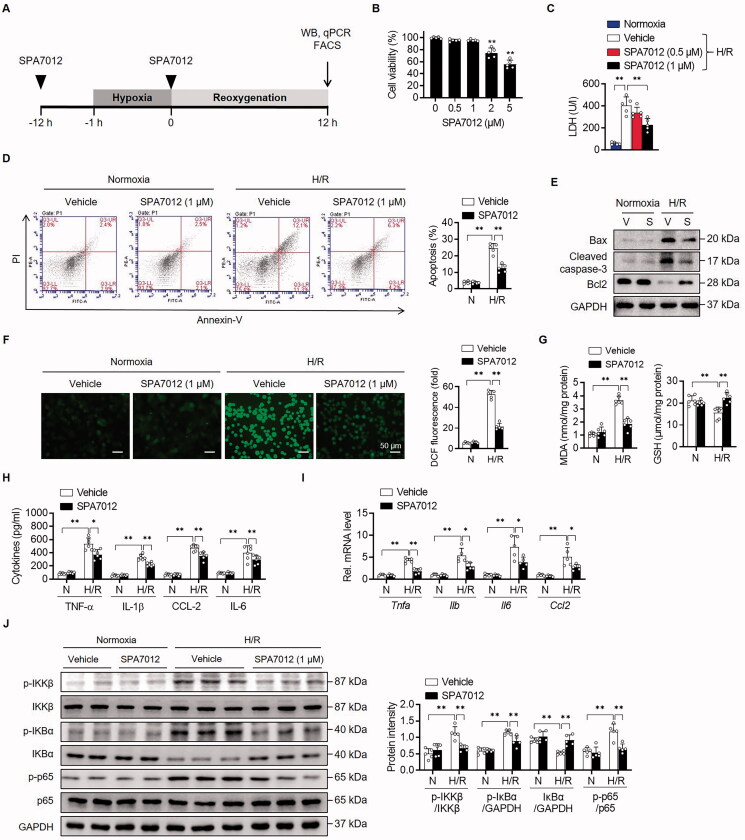
**Attenuation of H/R-induced apoptotic cell death and cytokine production by SPA7012.** (A) Schematic diagram of treatment of SPA7012 to primary hepatocytes. (B) MTT assay for cell viability (*n* = 5). (C) Lactate dehydrogenase (LDH) release from primary hepatocytes after 12 h reoxygenation (*n* = 5). (D and E) Analysis of apoptotic cell death by Annexin-V staining and Western blotting (*n* = 5). (F and G) Reactive oxygen species levels assessed using fluorescence microscopy following the changes in DCF fluorescence and colorimetric analysis of MDA and GSH (*n* = 5). (H and I) The protein and mRNA levels of pro-inflammatory cytokines/chemokine in culture media and hepatocytes (*n* = 5). (J) Protein levels of NF-κB signalling pathway (*n* = 5). Values are the mean ± SD. *p < 0.05 and **p < 0.01. H/R: hypoxia-reoxygenation.

In order to simulate hepatic I/R injury, we established an *in vitro* H/R-induced hepatocellular damage model (Figure 5(A)). SPA7012 at a concentration of less than 1 μM had little effect on cell viability (Figure 5(B)). Exposure to H/R significantly increased LDH release from primary hepatocytes compared with the normoxic group. However, when the cells were cultured with SPA7012 at 1 μM concentration before exposure to reoxygenation injury, cell damage was markedly decreased compared to only H/R (Figure 5(C)). Based on these results, 1 μM of SPA7012 was selected for the following experiments. Similar to I/R injury, H/R injury increased apoptotic cell death (Figure 5(D,E)), oxidative stress (Figure 5(F,G)), and cytokine release in primary hepatocytes (Figure 5(H,I)), while SPA7012 treatment resulted in a decrease of these negative responses. Again, SPA7012 treatment decreased phosphorylation levels of IKKβ and p65, and increased protein levels of IκBα compared to control group (Figure 5(J)).

## 4. Discussion

In the present study, we showed the hepatoprotective effects of PAK4 inhibitor SPA7012 in a mouse model of hepatic I/R. This protection confirms the results of our previous study^9^ and is attributed to Nrf2 protein stabilisation by PAK4 inhibitor and its anti-inflammatory effect.

Through *in silico* fragment screening, privileged bicyclic adenine pocket-binding scaffolds were selected to secure PAK4 specific inhibitors. Novel pyrazolo[3,4-*d*]pyrimidine derivatives with an allosteric back pocket binder were discovered as a type I ½ inhibitor, while the aforementioned CZh-226 was classified as a type I inhibitor. The type I inhibitors bind to the adenine site of the ATP pocket, with the Asp-Phe-Gly (DFG) motif of the activation loop adopting an ‘in’ conformation. Type II inhibitors bind to both the adenine site and an adjacent allosteric pocket created by accepting a DFG ‘out’ conformation. It is subdivided into the type I ½ inhibitors, which bind to an inactive kinase with a DFG ‘in’ structure.^21^ For kinome selectivity, type I ½ inhibitors are advantageous.^22^ Based on the number of identified kinase targets, type I ½ inhibitors indicate higher target selectivity (69%) compared to type I inhibitors (35%). Among the synthesised compounds, a pyrazolo[3,4-*d*]pyrimidine compound SPA7012 showed successful potency with an IC_50_ value of 0.77 μM and PAK4 selectivity.

During the process of hepatic I/R injury, redox homeostasis is impaired and large quantities of ROS are produced, resulting in protein and lipid peroxidation and DNA damage. Studies have demonstrated that the activation of Nrf2 is beneficial in maintaining integrity and function of liver tissues.^4^^,^^23^ Our recent study uncovered the regulation of Nrf2 protein stability by PAK4. Upon hepatic I/R injury, PAK4 is transcriptionally activated via HIF-1α. The induced PAK4 directly phosphorylates Nrf2 at T369, which leads to the nuclear export and proteasomal degradation of Nrf2. In contrast, genetic ablation of *Pak4* in hepatocytes stabilises Nrf2 and induces antioxidant enzymes such as HO-1 and NQO1, preventing oxidative damage.^9^ In the current study, pre-treatment of mice twice with SPA7012 at 50 mg/kg considerably increased post I/R protein levels of total-and nuclear-Nrf2 as well as its target genes in liver tissues. The subject study indicated that SPA7012 treatment resulted in reduced oxidative injury in I/R exposed mice liver.

The phosphorylation of Nrf2 is also regulated by several other kinases. The best characterised regulator is the PI3K-Akt-GSK-3β pathway as GSK-3β phosphorylates Nrf2, resulting in its nuclear exclusion and functional inhibition.^7^ Akt phosphorylates and thus inhibits GSK-3β activity, positively regulating Nrf2.^24^ In addition, PI3K/Akt blockade impedes Nrf2 transcriptional activity and aggravates I/R damage.^25^ Recently, we reported PAK4 phosphorylates PPARγ as capable of inducing PTEN, a lipid phosphatase that opposes the action of the PI3K-Akt pathway in myotoxin-treated myocytes.^26^ Thus, the question arises as to whether Nrf2 transcriptional activation is affected by PI3K-Akt-GSK-3β signalling in our model. Our results showed no changes to total- and phospho-protein levels of PPARγ, PTEN, Akt, and GSK-3β following SPA7012 treatment (data not shown), indicating that Nrf2 activation and concomitant hepato-protection by SPA7012 in I/R injury are not related to the Akt/GSK-3β dependent pathway.

We found in this study that SPA7012 treatment markedly suppressed canonical NF-κB signalling, as it reduced the phosphorylation of IKKβ/p65 and the transcription of pro-inflammatory cytokines and adhesion molecules. This was coupled with a drastic reduction of macrophage and neutrophil infiltration in post-ischemic liver tissues. Given that NF-κB plays a critical role in regulating inflammation, oxidative stress, cell death, and cell survival,^27^ we suggest that inhibition of NF-κB signalling by SPA7012 may represent a significant role in protection from I/R injury. Related to these findings, PAK4 is known to activate canonical NF-κB signalling (essentially via RELA, NF-κB p65 subunit) in cultured cancer cells,^28–30^ which contribute to cell proliferation and cell migration. Although we do not provide the direct evidence that SPA7012 inhibited NF-κB signalling through PAK4 inhibition, this study strongly suggests that PAK4 inhibition exerts an anti-inflammatory property during hepatic I/R.

PAK4 has been shown to promote cell proliferation and suppress apoptosis in many cancer models.^28^^,^^30^^,^^31^ However this conclusion is not in agreement with our model, where PAK4 inhibition resulted in strong anti-apoptotic effects, as evidenced by a decreased number of TUNEL-positive cells with concomitant decreased hepatic levels of pro-apoptotic proteins. A causal link may exist between NF-κB activation and the reduction in apoptotic cell numbers in hepatic tissue, as NF-κB activation has been considered a key process involved in apoptotic cell death during reperfusion.^32^ These results concur with our recent study showing that the *Pak4* deletion in hepatocytes suppresses both NF-κB and TUNEL-positive cells in liver tissues.^9^ Similarly, our previous report also shows that NF-κB suppression by A20 decreases apoptotic cell numbers in liver tissues after hepatic I/R.^15^ Thus, the inhibition of PAK4 has the ability to suppress apoptotic cell death following hepatic I/R.

In summary, this study demonstrates that pharmacological inhibition of PAK4 by SPA7012 ameliorates hepatic I/R injury in mice, which occurs as a result of stabilising Nrf2 and enhancing antioxidant capacity. Additionally, these results reveal the anti-apoptotic and anti-inflammatory properties of SPA7012 in liver tissues. These findings can be used to broaden our understanding of PAK4 on the pathogenesis of hepatic I/R, and support PAK4 as a new molecular target for therapeutic approaches to remedy I/R injuries in another organs.

## Abbreviations

I/R: ischaemia–reperfusion
H/R: hypoxia-reoxygenation
PAK4: p21-activated kinase 4
Nrf2: nuclear factor-erythroid 2-related factor 2
Keap1: Kelch-like ECH-associated protein 1
HO-1: haem oxygenase-1
NQO1: NAD(P)H quinone oxidoreductase 1
GPx: glutathione peroxidase
ALT: alanine aminotransferase
AST: aspartate aminotransferase
GSH: glutathione
MAD: malondialdehyde
LDH: lactate dehydrogenase
Co-IP: co-immunoprecipitation
